# Surface Roughness and Color Stability of Conventional Glass Ionomer Cement Reinforced With a Nanofiller Synthesized by the Coprecipitation Method

**DOI:** 10.1155/ijbm/4952239

**Published:** 2026-04-30

**Authors:** Neven S. Aref

**Affiliations:** ^1^ Department of Basic Oral Sciences and Dental Education, College of Dentistry, Qassim University, Buraydah, Saudi Arabia, qu.edu.sa; ^2^ Department of Dental Biomaterials, Faculty of Dentistry, Mansoura University, Mansoura, Egypt, mans.edu.eg

**Keywords:** chitosan, color stability, glass ionomer cement, hydroxyapatite, nanocomposite, surface roughness

## Abstract

**Aim:**

Modification of conventional glass ionomer cement (GIC) using nanofillers is a promising approach to enhance its clinical performance. This study aimed to evaluate the surface roughness and color stability of conventional GIC after modification with a synthesized 70:30 hydroxyapatite/chitosan (HA/CTS) nanocomposite.

**Materials and Methods:**

A 70:30 hydroxyapatite/chitosan nanocomposite was synthesized and incorporated into GIC powder at 1, 3, and 5 wt.%. Four groups were prepared: Group I (control), Group II (1 wt.% HA/CTS), Group III (3 wt.% HA/CTS), and Group IV (5 wt.% HA/CTS). A total of 80 specimens were fabricated (*n* = 10 per group for each test). Surface roughness (Ra) was measured using a profilometer, while color stability (ΔE) was assessed using a spectrophotometer. Data were analyzed using one‐way ANOVA followed by Tukey’s post hoc test (*α* = 0.05).

**Results:**

GIC modified with the 3 wt.% HA/CTS nanocomposite exhibited the lowest surface roughness and demonstrated clinically acceptable color change (ΔE ≤ 3.3) (*p* < 0.05). Higher nanocomposite loading (5 wt.%) resulted in significantly increased surface roughness and clinically perceptible color alteration.

**Conclusions:**

Incorporation of the 3 wt.% 70:30 HA/CTS nanocomposite into conventional GIC produced a smoother surface and acceptable esthetic outcome, suggesting a promising formulation for improved clinical performance.

## 1. Introduction

Glass ionomer cements (GICs) have been widely used because of their favorable properties, including direct adhesion to dental structures, a coefficient of thermal expansion comparable to that of natural tooth structure, biocompatibility, and prolonged fluoride release [1, 2]. Nevertheless, these materials have certain clinical drawbacks, including a relatively long setting reaction time, dehydration, moisture sensitivity during the initial setting stage, and a rough surface texture that may impair mechanical resistance [3].

There have been reports of amalgam, silver, and metal powders being added to GIC powder as reinforcements. Although the wear resistance is superior to that of the conventional GICs, the flexural strength and abrasion resistance are not significantly better, while the fluoride release is reduced. Because of the presence of a metallic phase, the cermet cements had exhibited inferior esthetics  [4]. Differences in compressive and flexural strength between conventional GIC and resin‐modified glass ionomer cements (RMGICs), which may influence their mechanical performance and clinical durability, have been reported [5]. The biological response to conventional GIC and RMGICs has been evaluated using various parameters, such as odontoblastic changes, inflammatory response, tertiary dentin formation, microbial detection, morphological alterations, and cellular characteristics including viability, cell number, and metabolic activity. In vital pulp therapy procedures, conventional GIC has been shown to exhibit lower cytotoxicity compared with RMGIC [6]. Therefore, improving conventional GIC has gained increasing interest as an alternative strategy to overcome the limitations of other modified formulations. Enhancing the properties of conventional GIC may provide better mechanical performance while maintaining its favorable biological behavior and clinical advantages [7].

Nanotechnology has significantly contributed to the advancement of dental materials and technologies, enabling the development of restorative materials with improved clinical performance. This progress has led to more effective treatment approaches, enhanced patient comfort, and improved overall treatment outcomes in dental practice [8]. Accordingly, incorporating nanotechnology into GICs has attracted considerable attention as a promising strategy to enhance their mechanical, biological, and esthetic properties.

Recently, the mechanical properties of GIC have been enhanced by the incorporation of nanoparticles such as nano‐TiO_2_, nanohydroxyapatite (nHA), nano‐SiO_2_, and nano‐ZrO_2_. This improvement is attributed to better particle distribution, increased surface area, and higher surface energy. Nevertheless, further investigations are still required to optimize the physicochemical performance of modified GIC formulations [9].

The use of nHA has attracted considerable attention in dentistry due to its superior mechanical, physical, and chemical properties. These nanoparticles have been incorporated into various dental formulations to support preventive and restorative oral healthcare. Numerous nHA‐based dentifrices, mouthwashes, and remineralizing agents have been developed to prevent dental caries, while nanocomposites and nanoceramics have been used for restoring tooth defects such as decay, fractures, and tooth loss [10]. The incorporation of hydroxyapatite into conventional GIC has been reported to enhance several beneficial properties. It exhibits excellent biocompatibility and ion‐releasing capability, along with a strong chemical affinity to tooth structure. Additionally, it promotes rapid early strength development, allowing high strength to be achieved within a short period. Importantly, the addition of hydroxyapatite does not interfere with the inherent fluoride‐releasing ability or the dentin‐bonding capacity of GIC [11–13].

Chitosan has also been widely utilized in dental nanomaterials to enhance mechanical integrity, support dentin matrix regeneration, and provide antimicrobial activity. It has been used for canal sealing, tissue regeneration, and localized drug delivery in periodontal therapy [14, 15]. Owing to its biocompatibility and bioactive properties, chitosan has been recognized as a promising biomaterial for periodontal regeneration and enamel repair strategies [16]. Chitosan‐modified GIC demonstrated enhanced fluoride release, improved flexural resistance, and favorable biological properties including high biocompatibility and hydrophilicity as findings of a preliminary study [17].

Surface roughness is considered an important factor influencing the clinical performance of restorative materials, as rough surfaces promote bacterial adhesion and biofilm formation, increasing the risk of plaque accumulation and gingival inflammation. In contrast, smoother surfaces reduce microbial retention and contribute to improved esthetic outcomes and restoration longevity [18–22].

Advances in material science and nanotechnology have facilitated the development of restorative materials with improved durability, bioactivity, and functional adaptability. Nanotechnology enables the manipulation of materials at the nanoscale, offering innovative opportunities to enhance the performance of dental biomaterials [23, 24]. The use of nanocomposites, which combine the advantages of multiple components to achieve synergistic effects, represents a promising approach for improving GIC properties [25].

Recently, experimental glass ionomer formulations incorporating chitosan, titanium, zirconium, and hydroxyapatite nanoparticles have demonstrated improvements in mechanical properties, biocompatibility, and bioactivity. Such multifunctional nanocomposite systems aim to overcome the inherent limitations of conventional GIC and may represent next‐generation restorative materials for clinical application [26].

Despite the growing body of research on nHA‐modified GIC, limited attention has been directed toward the synergistic integration of hydroxyapatite with biopolymers such as chitosan to simultaneously enhance biological interaction and physicochemical performance. Furthermore, few studies have concurrently evaluated surface roughness and color stability, despite their critical roles in bacterial adhesion, plaque accumulation, and long‐term esthetic success.

A previous investigation [27] demonstrated that incorporation of HA/CTS nanocomposites into conventional GIC can improve several mechanical properties such as compressive strength, surface hardness, and shear bond strength. However, the influence of this nanocomposite system on clinically relevant surface and esthetic properties of GIC has not been sufficiently investigated. In particular, surface roughness and color stability are important parameters that influence bacterial adhesion, plaque accumulation, and the long‐term esthetic performance of restorative materials. Therefore, further investigation of these properties is necessary to determine whether incorporation of HA/CTS nanocomposites can provide a more comprehensive improvement in the overall clinical performance of GIC.

Accordingly, this study aimed to investigate the synergistic effect of a synthesized 70:30 hydroxyapatite/chitosan nanocomposite incorporated into conventional GIC, with emphasis on surface roughness and color stability as key indicators of biological and esthetic clinical performance. The null hypothesis was that incorporation of the 70:30 HA/CTS nanocomposite into conventional GIC would not improve surface smoothness or esthetic outcomes.

## 2. Materials and Methods

### 2.1. Synthesis of HA/CTS Nanocomposite

Materials used in the study are listed in Table 1. The hydroxyapatite/chitosan nanocomposite was prepared by the coprecipitation method at a ratio of 70:30. A 3 wt.% chitosan aqueous solution was prepared by dissolving 3 g of chitosan powder in 100 mL of distilled water containing 1 wt.% acetic acid with continuous stirring for 5 h until a clear solution was obtained. Subsequently, 10 wt.% phosphoric acid solution was added to the mixture. The reagent quantities were adjusted to achieve a final HA/chitosan weight ratio of 70:30.

**TABLE 1:** Materials used in the study.

Materials	Form	Manufacturer	Batch number
IONO‐GEM	Powder/liquid	DENTAL COMPOSITE LTD. UK.	0911294
Chitosan	Powder	Sigma‐Aldrich Co., 3050 Spruce Street, St. Louis.	MKBD7240V
Calcium hydroxide	Powder	Loba Chemie Pvt. Ltd., Mumbai 400005. India.	G448108
Phosphoric acid	Liquid	Sigma‐Aldrich Co., 3050 Spruce Street, St. Louis.	SZBB1030V
Acetic acid	Liquid	Sigma‐Aldrich Co., 3050 Spruce Street, St. Louis.	SZBB1330V
Ethanol	Liquid	Sigma‐Aldrich Co., 3050 Spruce Street, St. Louis.	SZBB23080
Sodium hydroxide	Powder	Al‐Nasr company, Egypt.	2009/1

The chitosan/phosphoric acid solution was then added dropwise into a vigorously stirred 4% ethanol solution of calcium hydroxide. The pH was adjusted to approximately 10 using a 2 M NaOH solution. A burette was used to control the dropping rate at approximately 4 mL/min. After titration, the resulting slurry was maintained under continuous stirring for 24 h and then aged for an additional 24 h. The precipitate was filtered, washed with deionized water, and dried at 80°C in a vacuum oven. The dried composite was ground using a ball milling machine (PM 400, Retsch, Germany) for 8 h at 300 rpm [28].

### 2.2. Characterization of the Nanocomposite

Characterization of the synthesized nanocomposite powder was performed using transmission electron microscopy (TEM; JEM‐2100, Japan) to verify nanoscale particle size. The powder was further analyzed using a Fourier transform infrared (FTIR) spectrometer (Nicolet iS10, Thermo Electron Corporation, UK) over a spectral range of 500–4000 cm^−1^.

### 2.3. Preparation of Modified GIC Powder

The HA/CTS nanocomposite (70:30) was incorporated into conventional GIC powder at concentrations of 1, 3, and 5 wt.%. The powders were manually blended using a glass slab and spatula, and then transferred into clean, empty amalgam capsules and vibrated for approximately 20 s in an amalgamator (Softly 8, de Gotzen, Italy) to ensure uniform dispersion.

### 2.4. Specimen Preparation

A total of 80 specimens were prepared: 40 for surface roughness testing and 40 for color stability assessment (*n* = 10 per group for each test). Specimens were prepared according to the following groups:

• Group I: control (conventional GIC)
• Group II: 1 wt.% HA/CTS‐modified GIC
• Group III: 3 wt.% HA/CTS‐modified GIC
• Group IV: 5 wt.% HA/CTS‐modified GIC

Specimens were prepared separately for each experimental group according to the designated nanocomposite concentration. All specimens were prepared under standardized conditions to ensure consistency among groups. Prepared specimens were stored in distilled water at 37°C for 48 h prior to testing.

### 2.5. Surface Roughness

Disc‐shaped specimens (8 × 2 mm) were used for surface roughness evaluation. Surface roughness was measured using a profilometer (Surftest SJ‐211, Mitutoyo, Tokyo, Japan). Five measurements were obtained from each specimen at points at least 0.5 mm apart and 1 mm from the specimen edge.

The stylus traversed a distance of 3.0 mm for each measurement with a cutoff value of 0.8 mm. The diamond stylus tip radius was 5 μm. Measurements were performed under controlled laboratory conditions with a measuring force of 4 mN and a speed of 0.5 mm/s. The average surface roughness (Ra) for each specimen was calculated as the mean of the five readings and expressed in μm. Measurements were performed in a blinded manner with respect to group identity to minimize observational bias.

### 2.6. Spectrophotometric Analysis

Disc‐shaped specimens (8 × 2 mm) were fabricated using a split Teflon mold. A spectrophotometer (VITA Easyshade, Vita Zahnfabrik H. Rauter GmbH & Co. KG, Bad Säckingen, Germany) was used to measure color coordinates (*L* ^∗^, *a* ^∗^, and *b* ^∗^).

The CIE *L* ^∗^, *a* ^∗^, and *b* ^∗^ values of each specimen were compared with those of the control group, and color difference (ΔE) was calculated using the following formula:

(1) ΔE=ΔL∗2+Δa∗2+Δb∗212/,

where *L* ^∗^ represents lightness, and *a* ^∗^ and *b* ^∗^ represent chromaticity coordinates. Color difference values (ΔE) ≤ 3.3 were considered clinically acceptable.

### 2.7. Statistical Analysis

Power Analysis and Sample Size Software (PASS, NCSS, LLC, Utah, USA) was used to determine the sample size. The study design provided 80% power at a significance level of 5% with an effect size of 0.4. Data normality was verified using the Shapiro–Wilk test. Statistical analysis was performed using SPSS Version 25 (SPSS Inc., Chicago, IL, USA). Data were analyzed using one‐way analysis of variance (ANOVA) followed by Tukey’s post hoc test at a significance level of α = 0.05.

## 3. Results

### 3.1. Characterization of the Prepared Nanocomposite

#### 3.1.1. TEM

**FIGURE 1:**
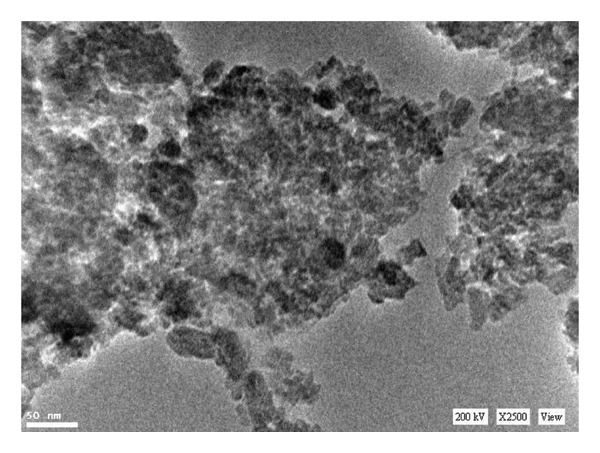
Transmission electron microscopy (TEM) image of the synthesized 70:30 hydroxyapatite/chitosan (HA/CTS) nanocomposite showing shuttle‐like particles approximately 80 nm in length and 20 nm in width.

The transmission electron micrograph of the synthesized nanocomposite is shown in Figure 1. Chitosan and hydroxyapatite precipitated to form a HA/CTS composite when the chitosan/H_3_PO_4_ solution was introduced dropwise into the highly alkaline Ca(OH)_2_ suspension. The crystals of the HA/CTS nanopowder exhibited a thin, shuttle‐like morphology, typically measuring approximately 80 nm in length and 20 nm in width, confirming the nanoscale structure of the synthesized composite.

#### 3.1.2. FTIR Spectroscopy

**FIGURE 2:**
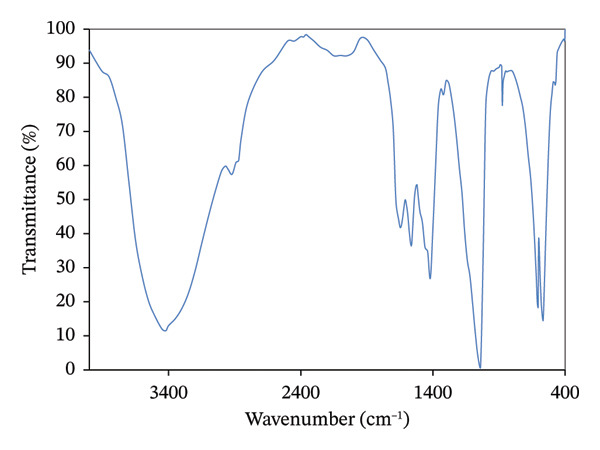
Fourier transform infrared (FTIR) spectrum of the synthesized 70:30 hydroxyapatite/chitosan (HA/CTS) nanocomposite showing characteristic phosphate bands at 500–600 and 1000–1100 cm^−1^, carbonate bands at 1420–1485 cm^−1^, and hydroxyl/amide bands at 1550–1700 cm^−1^.

The FTIR spectrum of the synthesized nanocomposite is presented in Figure 2. The phosphate bending bands were observed at 500–600 cm^−1^, while the characteristic hydroxyapatite phosphate stretching bands appeared at 1000–1100 cm^−1^. Broadening of the band near 1050 cm^−1^ indicates the presence of the polymer and its interaction with phosphate groups [28].

Bands observed at 1420–1485 cm^−1^ and approximately 875 cm^−1^ correspond to carbonate ions, whereas bands in the range of 2800–2950 and 3600–3700 cm^−1^ are attributed to hydroxyl groups in chitosan [29]. The bands between 1550 and 1700 cm^−1^ are associated with the superposition of hydroxyapatite OH groups and chitosan amide I and II bands [30].

The progressive interaction between Ca^2+^ ions of hydroxyapatite and the NH_2_ functional groups of chitosan likely contributed to these spectral variations, indicating successful chemical interaction and bonding between chitosan and hydroxyapatite [31].

### 3.2. Surface Roughness (μm)

**FIGURE 3:**
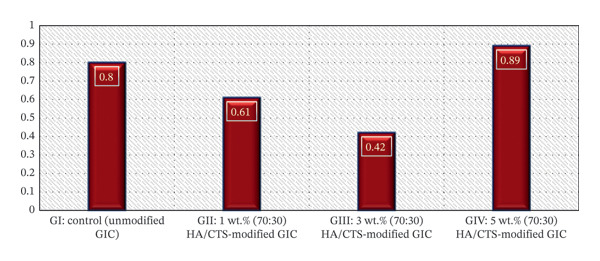
Mean surface roughness (Ra, μm) of conventional glass ionomer cement unmodified and modified with the 70:30 hydroxyapatite/chitosan (HA/CTS) nanocomposite at concentrations of 1, 3, and 5 wt.%.

Means, standard deviations, and Tukey’s post hoc analysis of surface roughness are presented in Table 2. The lowest mean surface roughness value was observed in the 3 wt.% HA/CTS‐modified GIC group (0.42 ± 0.27), whereas the 5 wt.% HA/CTS group exhibited the highest value (0.89 ± 0.33). One‐way ANOVA revealed a statistically significant difference among groups (*p* < 0.05). Tukey’s post hoc analysis demonstrated that both Group I (control) and Group IV (5 wt.% HA/CTS‐modified GIC) differed significantly from Group III (3 wt.% HA/CTS‐modified GIC) (*p* < 0.05). Group II (1wt.% HA/CTS modified GIC) showed no statistically significant difference with any of the other groups (*p* < 0.05). A graphical presentation of the surface roughness results is presented in Figure 3.

**TABLE 2:** Mean surface roughness (μm) of the studied groups.

Groups	Surface roughness (*μ*m)
	Mean ± SD
I Control (unmodified GIC)	0.80^a^ ± 0.09
II 1 wt.% (70:30) HA/CTS‐modified GIC	0.61^ab^ ± 0.13
III 3 wt.% (70:30) HA/CTS‐modified GIC	0.42^b^ ± 0.27
IV 5 wt.% (70:30) HA/CTS‐modified GIC	0.89^a^ ± 0.33
*p* value	< 0.001

*Note:* Means with the same superscript letter are not significantly different at *p* ≤ 0.05.

### 3.3. Spectrophotometric Assessment

**FIGURE 4:**
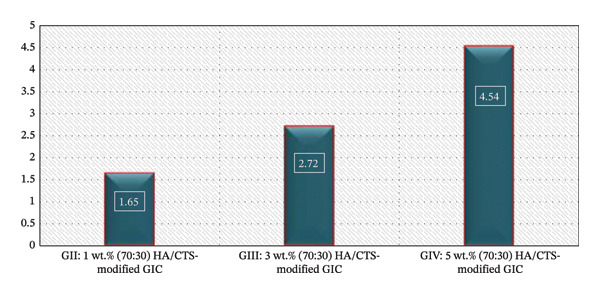
Mean color difference (ΔE) of conventional glass ionomer cement modified with 70:30 hydroxyapatite/chitosan (HA/CTS) nanocomposite at concentrations of 1, 3, and 5 wt.% relative to the unmodified control group. The clinical acceptability threshold (ΔE = 3.3).

Table 3 presents the ANOVA and Tukey’s post hoc results for color stability. GIC modified with the 5 wt.% HA/CTS nanocomposite showed the highest mean color difference (4.54 ± 0.83), whereas the 1 wt.% HA/CTS group exhibited the lowest value (1.65 ± 0.23). One‐way ANOVA indicated a significant difference among groups (*p* < 0.05), with all groups differing significantly from each other. Figure 4 shows the color variations among the tested groups.

**TABLE 3:** Mean color difference (ΔE) of the studied groups.

Groups	Color difference (ΔE)
	Mean ± SD
II 1 wt.% (70:30) HA/CTS‐modified GIC	1.65^c^ ± 0.23
III 3 wt.% (70:30) HA/CTS‐modified GIC	2.72^b^ ± 0.17
IV 5 wt.% (70:30) HA/CTS‐modified GIC	4.54^a^ ± 0.83
*p* value	< 0.001

*Note:* Means with the same superscript letter are not significantly different at *p* ≤ 0.05. ΔE values represent color change relative to the control group.

Modification of GIC with 1 and 3 wt.% HA/CTS produced clinically acceptable color changes (ΔE = 1.65 ± 0.23 and 2.72 ± 0.17, respectively; ΔE ≤ 3.3), whereas 5 wt.% HA/CTS‐modified GIC exhibited a perceptible color change (ΔE > 3.3).

## 4. Discussion

In a previous study, the 70:30 HA/CTS nanocomposite demonstrated enhanced compressive strength, shear bond strength to tooth structure, and surface microhardness of conventional GIC, exhibiting superior performance among the investigated HA/CTS ratios [27]. In the present study, the 70:30 HA/CTS ratio was selected based on previous findings indicating enhanced chemical and mechanical interlocking, hydrogen bonding between NH_2_ and OH groups, and chelation between NH_2_ groups and calcium ions, which collectively improve interfacial interaction and structural integrity [32].

Surface roughness is a critical property of GIC, as rough surfaces may enhance plaque accumulation, bacterial adhesion, and compromise the esthetics and longevity of restorations [18]. Surface roughness values exceeding approximately 0.2 μm have been reported to facilitate bacterial adhesion and biofilm retention, highlighting the clinical importance of achieving smoother restorative surfaces. The null hypothesis was rejected, as modification of GIC with the synthesized 70:30 HA/CTS nanocomposite produced significant changes in both surface roughness and color stability. The 3 wt.% concentration produced a smoother surface compared with 1 wt.%, whereas 5 wt.% increased surface roughness, although the increase remained nonsignificant.

The incorporation of the nanocomposite at low to moderate concentrations (1 and 3 wt.%) improved surface smoothness through filling microvoids and improving particle packing within the cement matrix [33, 34]. The improved smoothness observed at 3 wt.% HA/CTS suggests an optimal balance between filler dispersion and matrix integration. At this concentration, nanoparticles are more likely to distribute uniformly and reinforce the cement matrix without significant agglomeration. The findings of Sharafeddin and Bahrani support this trend, demonstrating that optimal nHA concentrations enhance GIC surface quality by improving particle packing and reducing microvoids, thereby producing a smoother surface finish. Additionally, nHA particles can reduce surface imperfections by penetrating interstitial spaces between glass particles due to their small size and large surface area [34].

Conversely, increasing the nanocomposite concentration to 5 wt.% resulted in a slight increase in surface roughness. This may be attributed to particle agglomeration and uneven dispersion, leading to surface irregularities [35]. Excessive filler loading can disrupt matrix continuity and expose filler clusters, contributing to increased roughness. Similar findings have been reported in HA/collagen nanocomposite–modified GIC systems, where higher filler content resulted in surface discontinuities and increased roughness [36].

With reference to the role of chitosan, its incorporation may also influence surface characteristics. In addition to enhancing mechanical properties and interfacial bonding, chitosan can affect matrix organization depending on its concentration. A previous study has shown that chitosan‐modified GIC exhibits clinically acceptable roughness; however, its effect on surface topography varies with formulation and environmental conditions, suggesting that biopolymer interactions within the cement matrix can modulate surface characteristics [37].

Collectively, these findings emphasize that optimizing nanocomposite filler loading is essential for improving GIC surface quality. While excessive loading may lead to surface deformities due to particle agglomeration, the low roughness observed at 3 wt.% HA/CTS suggests an effective balance between structural reinforcement and homogeneous nanoparticle dispersion. This pattern is consistent with findings in other modified GIC systems, where surface quality improved with moderate filler incorporation but deteriorated at higher concentrations [34].

Regarding spectrophotometric findings, the present study demonstrated a relationship between surface roughness and color stability. In addition to exhibiting the lowest surface roughness, the 3 wt.% HA/CTS group showed a clinically acceptable color change (ΔE < 3.3). Smoother surfaces promote more uniform light reflection and reduce light scattering, thereby enhancing color stability and esthetic appearance [18, 38].

Conversely, the significantly higher ΔE observed in the 5 wt.% HA/CTS group may be attributed to increased filler loading, which can negatively affect the optical properties of the cement. Particle agglomeration, interfacial mismatch, and disruption of matrix homogeneity increase light scattering and reduce translucency. These findings are consistent with reports indicating that excessive filler content and particle clustering adversely affect the optical properties and color stability of restorative materials [38]. The present findings are consistent with a previous report indicating that the incorporation of (chitosan–Ti–Zr–HA) nanocomposite fillers into GIC can enhance its esthetic properties, including color stability and surface gloss. However, these improvements appear to be concentration‐dependent, as the amount of filler loading plays a critical role in determining the final esthetic performance of the material [39]. Additionally, other studies evaluating the modification of GIC with nanoparticles (carbon nanotubes and silver nanoparticles) have reported variable esthetic outcomes. Although carbon nanotubes demonstrated better color stability than silver nanoparticles, both modified groups showed lower color stability than the unmodified GIC [40].

From a clinical perspective, the smoother surface achieved with 3 wt.% HA/CTS modification may reduce bacterial adhesion, plaque accumulation, and gingival inflammation while maintaining acceptable esthetic outcomes. These characteristics are essential for improving restoration longevity and patient satisfaction.

### 4.1. Study Limitations

Despite these promising findings, this study has limitations. It was conducted under in vitro conditions that do not fully replicate the complex oral environment, including masticatory forces, thermal fluctuations, pH variations, and long‐term aging. Future investigations should evaluate additional properties such as fluoride release, wear resistance, and long‐term clinical performance to confirm the clinical applicability of this modification.

## 5. Conclusions

Within the limitations of this study, incorporation of the 3 wt.% of a 70:30 hydroxyapatite/chitosan nanocomposite into conventional GIC improved surface smoothness while maintaining clinically acceptable color stability. This modification may contribute to reduced plaque retention, improved esthetics, and enhanced restoration longevity. In contrast, higher nanocomposite loading (5 wt.%) negatively affected both surface roughness and color stability. Therefore, careful optimization of nanocomposite concentration is essential for achieving balanced biological and esthetic performance in modified GIC formulations.

## Author Contributions

The author, Neven S. Aref, was alone responsible for the conception, study design, investigation and execution, data acquisition, analysis, and interpretation; the article’s drafting, revision, or critical review.

## Funding

There is no funding to report. The author did not receive financial support from any organization for the submitted work.

## Disclosure

The author gave final approval of the version to be published and agreed to be accountable for all aspects of the work,

## Ethics Statement

This research did not involve any human subjects or animal specimens, and it was performed in accordance with the institutional regulations.

## Consent

The author has nothing to report.

## Conflicts of Interest

The author declares no conflicts of interest.

## Data Availability

The data that support the findings of this study are available from the corresponding author upon reasonable request.
